# Hesperidin Suppresses the Proliferation of Prostate Cancer Cells by Inducing Oxidative Stress and Disrupting Ca^2+^ Homeostasis

**DOI:** 10.3390/antiox11091633

**Published:** 2022-08-23

**Authors:** Seon Ae Jeong, Changwon Yang, Jisoo Song, Gwonhwa Song, Wooyoung Jeong, Whasun Lim

**Affiliations:** 1 Department of Biological Sciences, College of Science, Sungkyunkwan University, Suwon 16419, Korea; 2 Institute of Animal Molecular Biotechnology, Department of Biotechnology, College of Life Sciences and Biotechnology, Korea University, Seoul 02841, Korea; 3 Department of Biomedical Sciences, Catholic Kwandong University, Gangneung 25601, Korea

**Keywords:** prostate cancer, hesperidin, proliferation, Ca^2+^ homeostasis, flavonoid

## Abstract

Although androgen deprivation therapy is mainly used for its treatment, the mortality rate of prostate cancer remains high due to drug resistance. Hence, there is a need to discover new compounds that exhibit therapeutic effects against prostate cancer with minimum side effects. Hesperidin is a flavonoid carbohydrate isolated from citrus fruits. It has antiproliferative effects in various cancer types; however, whether it can modulate cell proliferation by modulating the key targets of cancer therapy, including intracellular signaling pathways and oxidative stress, remains unknown. Therefore, we confirmed that hesperidin suppressed the proliferation of prostate cancer cells, PC3 and DU145. Hesperidin induced cell death by regulating the cell cycle and inhibited the expression of proliferating cell nuclear antigen, a cell proliferation marker. Hesperidin also promoted the generation of reactive oxygen species and induced mitochondrial membrane depolarization and endoplasmic reticulum stress in prostate cancer cells. Moreover, as hesperidin increased Ca^2+^ levels in prostate cancer cells, we co-treated the inositol 1,4,5-trisphosphate receptor inhibitor, 2-aminoethyl diphenyl borate (2-APB), with hesperidin. Notably, 2-APB restored cell proliferation, which was reduced to control levels by hesperidin. In addition, hesperidin inhibited the activation of the phosphoinositide 3-kinase and mitogen-activated protein kinase signaling pathways. Hesperidin also enhanced the anticancer effects of the chemotherapeutic agent, cisplatin, in both PC3 and DU145 cells. Taken together, these results suggest that hesperidin can be used as a potential therapeutic adjuvant in prostate cancer as it can inhibit cell proliferation by mediating oxidative stress and increasing Ca^2+^ levels.

## 1. Introduction

Prostate cancer has the third highest mortality rate worldwide and is the second most common cancer in men [1,2]. Androgens are critical factors that promote the development of prostate cancer, and androgen deprivation therapy (ADT) is the primary strategy for prostate cancer treatment [3]. ADT drugs include inhibitors of androgen synthesis and androgen receptor activation [4]. However, continuous ADT results in castration-resistant prostate cancer, with an increased risk of mortality [5]. In this regard, development of therapeutic adjuvants with fewer side effects to complement traditional treatment methods for prostate cancer is important [6,7].

Phytochemicals are excellent for cancer treatment because of their low side effects, easy extraction from plants, and cost benefits [8]. Several epidemiological studies have reported a negative correlation between phytochemical-rich diet and risk of various cancer types, including breast, colon, lung, pancreatic, and prostate cancers [9,10]. Hesperidin, a flavanone glycoside, is effective against various cancer types, including oral [11], breast [12,13] and lung cancers [14]. In previous reports, hesperidin regulated the cell cycle and endoplasmic reticulum (ER) stress, leading to apoptosis [15,16]. Previous studies conducted on prostate cancer have reported conflicting results regarding the effect of hesperidin on the proliferative capacity of prostate cancer [17,18]. Therefore, we aimed to elucidate the role of hesperidin in regulating the proliferative capacity of prostate cancer cells and the related intracellular mechanisms.

Evidence from clinical studies suggests that intracellular mechanisms, including activation of the phosphoinositide 3-kinase (PI3K) signaling pathway and disruption of oxidative homeostasis, contribute to castration-resistant prostate cancer [19,20]. The PI3K pathway can also interact with the mitogen-activated protein kinase (MAPK) pathway, which contributes to the proliferation, survival, and drug resistance of prostate cancer cells [21,22]. In addition, the induction of excessive oxidative stress via the application of external factors is an important anticancer strategy for inducing apoptosis by exceeding the elevated stress threshold in cancer cells compared with normal cells. The induction of oxidative stress is associated with the disruption in mitochondrial membrane potential and increased intracellular Ca^2+^ levels following ER stress in cancer cells [23,24]. Moreover, intracellular Ca^2+^ activity regulates cell proliferation, migration, and death; therefore, store operated Ca^2+^ entry (SOCE)-related factors, including the pore-forming Ca^2+^ channel subunit, are targets for anticancer effects [25]. However, it is unclear whether hesperidin suppresses cell proliferation by regulating oxidative stress and Ca^2+^ flux in prostate cancer. Therefore, we estimated the physiological effects related to the anticancer action of hesperidin in PC3 and DU145 cells.

## 2. Materials and Methods

### 2.1. Chemicals

Hesperidin was purchased from Sigma-Aldrich and dissolved in dimethyl sulfoxide. Antibodies against phosphorylated AKT, P70S6K, ERK1/2, P90RSK, P38, eIF2α, and total AKT, P70S6K, ERK1/2, P90RSK, P38, IRE1α, and eIF2α were purchased from Cell Signaling Technology (Beverly, MA, USA). Antibody against ATF6α was purchased from Santa Cruz Biotechnology (Dallas, TX, USA). LY294002 was purchased from Cell Signaling Technology (Beverly, MA, USA). U0126 and SB203580 were purchased from Enzo Life Sciences (Farmingdale, NY, USA). Cisplatin and paclitaxel were purchased from Sigma-Aldrich (St.Louis, MO, USA).

### 2.2. Cell Culture

PC3 and DU145 cells were purchased from the American Type Culture Collection (Manassas, VA, USA). These cell lines were cultured in Roswell Park Memorial Institute-1640 medium supplemented with 25 mM HEPES, 10% fetal bovine serum (FBS), and 1% penicillin/streptomycin (100 U/mL). The cells were then incubated at 37 °C in a 5% CO_2_ incubator. The cells were then treated with different concentrations of hesperidin.

### 2.3. BrdU Incorporation Analysis for Cell Proliferation Measurements

Cell proliferation was measured using a Cell Proliferation ELISA BrdU kit purchased from Roche (Basel, Switzerland). Cells were labeled with BrdU and incubated for 2 h. Cells were then fixed and incubated with anti-BrdU-peroxidase, which reacted with the substrate 3,3′,5,5′-tetramethylbenzidine substrate to produce an immune complex. The product was measured using an ELISA reader at wavelengths of 370 and 490 nm.

### 2.4. Spheroid Formation

Spheroids were formed by modifying the hanging drop method, as described previously [7]. Briefly, 12,000 cells seeded in the lids of the culture dishes were grown for three days with 20 μM hesperidin at 37 °C in a 5% CO_2_ incubator. Changes in spheroid morphology were observed using a DM3000 microscope (Leica, Wetzlar, Germany), and the relative density values were calculated using the ImageJ software (1.53v, NIH).

### 2.5. Quantitative Real-Time Polymerase Chain Reaction (qPCR)

RNA extracted after treatment with hesperidin for 24 h was used for the synthesis of complementary DNA (cDNA) using Oligo dT, random primers, and AccuPower PreMix (Bioneer, Daejeon, Korea), as described in the manufacturer’s manual. Differences in gene expression were estimated via qPCR using SYBR dye. Fluorescence intensity was analyzed as reported in previous studies [26,27]. The primer sets, used for qPCR, are listed in Table 1.

### 2.6. Intracellular Ca^2+^ Measurements

Intracellular Ca^2+^ levels were analyzed as described in a previous study [7]. Briefly, cells were harvested and fluorescence intensity was estimated using 3 μM fluo-4 (Invitrogen, Carlsbad, CA, USA). The fluorescence intensity representing the Ca^2+^ concentration was determined using a flow cytometer.

### 2.7. Cellular ROS Measurements

Cellular ROS levels were analyzed as described in a previous study [7]. Briefly, cells were collected and stained with 2′,7′-dichlorofluorescein diacetate (DCFH-DA) (Sigma-Aldrich, St.Louis, MO, USA). 2′,7′-dichlorofluorescein (DCF) fluorescence intensity was analyzed using flow cytometry, as described in a previous study.

### 2.8. Cell Cycle Analysis

Collected cells were incubated with RNase A (Sigma-Aldrich) and propidium iodide (BD Biosciences, Franklin Lakes, NJ, USA). Subsequently, fluorescence was detected using a flow cytometer, and the results were analyzed by dividing the cells into the subG1, G0/G1, S, and G2/M phases.

### 2.9. Mitochondrial Membrane Potential (MMP) Assay

After seeding 2 × 10^5^ cells into a 6-well plate, they underwent dose-dependent treatment with hesperidin (0, 5, 10, and 20 μM). The collected cells were incubated with the JC-1 dye at 37 °C for 20 min. After incubation, the cells were washed with PBS and the fluorescence intensity was analyzed using a flow cytometer.

### 2.10. Western Blotting

Bradford assay was performed using the BSA standard for protein concentration estimation. After denaturing the proteins, sodium dodecyl sulfate-polyacrylamide gel electrophoresis was performed to separate the proteins based on their size. The proteins were then transferred to nitrocellulose membranes. After attaching the desired target protein to the membrane through an antigen–antibody reaction, the light intensity of the immunoblots was measured using the ChemiDoc EQ system (Bio-Rad, Hercules, CA, USA).

### 2.11. Apoptosis Analysis

Cell apoptosis was analyzed using an Annexin V Apoptosis Detection Kit I (BD Biosciences, Franklin Lakes, NJ, USA). After seeding 2 × 10^5^ cells into a 6-well plate, they underwent dose-dependent treatment with hesperidin (0, 5, 10, and 20 μM) and co-treatment with cisplatin and paclitaxel. The collected cells were incubated with annexin V and propidium iodide (PI) for 15 min at room temperature. The fluorescence intensity was analyzed using a flow cytometer.

### 2.12. Statistical Analysis

All data were analyzed according to a general linear model using the SAS program to confirm statistical significance. Differences were considered statistically significant at *p* < 0.05. Statistical data were analyzed as the mean ± standard error unless otherwise noted.

## 3. Results

### 3.1. Hesperidin Decreases the Proliferation of Castration-Resistant Prostate Cancer Cells

**Figure 1:**
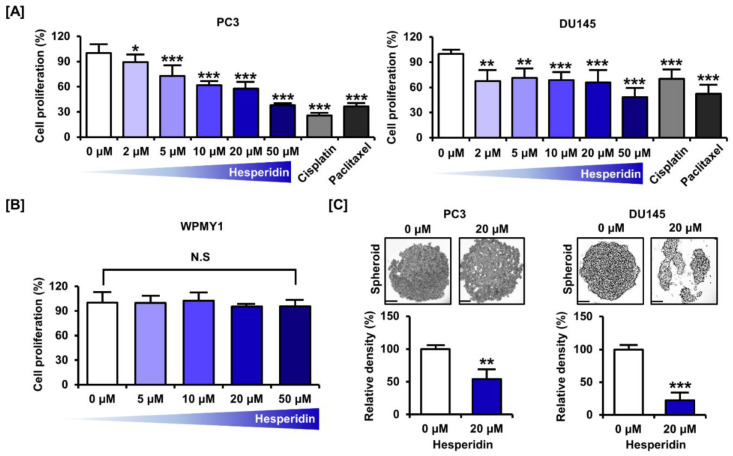
Effect of hesperidin on the proliferation of prostate cancer cells. (**A**) Relative proliferation of PC3 and DU145 cells was verified after dose-dependent treatment with hesperidin (0, 2, 5, 10, 20, and 50 μM), cisplatin (5 μM), and paclitaxel (5 μM) for 48 h. (**B**) Relative proliferation of WPMY1 cells was verified after dose-dependent treatment with hesperidin (0, 5, 10, 20, and 50 μM) for 48 h. (**C**) Effects of hesperidin on spheroid formation in PC3 and DU145 cells. Scale bar represents 300 μm. All results of the arithmetic mean (*n* = 3) denote statistical significance with asterisks (* *p* < 0.05, ** *p* < 0.01 and *** *p* < 0.001).

We confirmed the inhibition of proliferation in castration-resistant prostate cancer cells, PC3 and DU145, in response to dose-dependent treatment with hesperidin. The proliferative capacity decreased to 37.9% (*p* < 0.001) in PC3 cells and 48.4% (*p* < 0.001) in DU145 cells at the highest concentration of 50 µM (Figure 1A). In contrast, treatment with the anticancer drugs, cisplatin and paclitaxel, at 5 µM decreased the proliferation by 74.4% (*p* < 0.001) and 63.6% (*p* < 0.001), respectively, in PC3 cells and by 61.1% (*p* < 0.001) and 71.0% (*p* < 0.001), respectively, in DU145 cells. Moreover, the cytotoxicity of hesperidin was investigated in the normal prostate cell line, WPMY1. We found that hesperidin did not significantly affect the proliferation of WPMY1 cells (Figure 1B). Spheroid formation was analyzed after 3 days of culture using the hanging drop method in the lid of the culture dish. The relative density of tumor spheroids was reduced by 45.7% (*p* < 0.01) in PC3 cells and 77.6% (*p* < 0.001) in DU145 cells (Figure 1C). These results indicate that hesperidin suppresses prostate cancer cell proliferation.

### 3.2. Hesperidin Downregulates the Cell Proliferation Marker Expression in Prostate Cancer Cells

**Figure 2:**
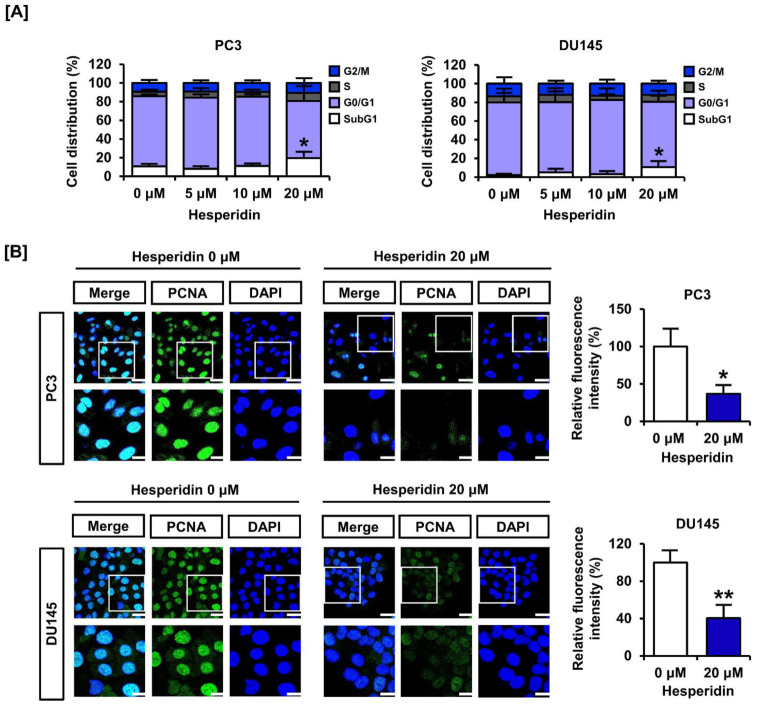
Effect of hesperidin on the cell cycle arrest of prostate cancer cells. (**A**) Cell cycle distribution was analyzed after dose-dependent treatment of PC3 and DU145 cells with hesperidin via flow cytometry. (**B**) The ability of hesperidin to regulate the expression of proliferating cell nuclear antigen (PCNA) was analyzed via immunofluorescence using Alexa 488 (green). Then, 4, 6-diamino-2-phenylindole (DAPI; blue) was used to stain the nuclei of PC3 and DU145 cells. Scale bars of the first horizonal panels are 40 μm, and scale bars of the second horizonal panels are 20 μm. All results of the arithmetic mean (*n* = 3) denote statistical significance with asterisks (* *p* < 0.05 and ** *p* < 0.01).

We analyzed cell cycle distribution and expression of the proliferating cell nuclear antigen (PCNA). In response to dose-dependent treatment with hesperidin (0, 5, 10, and 20 µM), the relative cell distribution of the subG1 phase gradually increased, implying entry into the apoptotic process (Figure 2A). The immunofluorescence intensity of PCNA (green) was reduced by treatment of PC3 and DU145 cells with 20 µM hesperidin (Figure 2B). The relative fluorescence intensity was reduced by more than 50% in both cell lines. These results indicate that hesperidin regulates cell cycle distribution and suppresses the expression of proliferation markers in prostate cancer cells.

### 3.3. Hesperidin Induces ROS Production and Interferes with Mitochondrial Homeostasis and ER Function in Prostate Cancer Cells

**Figure 3:**
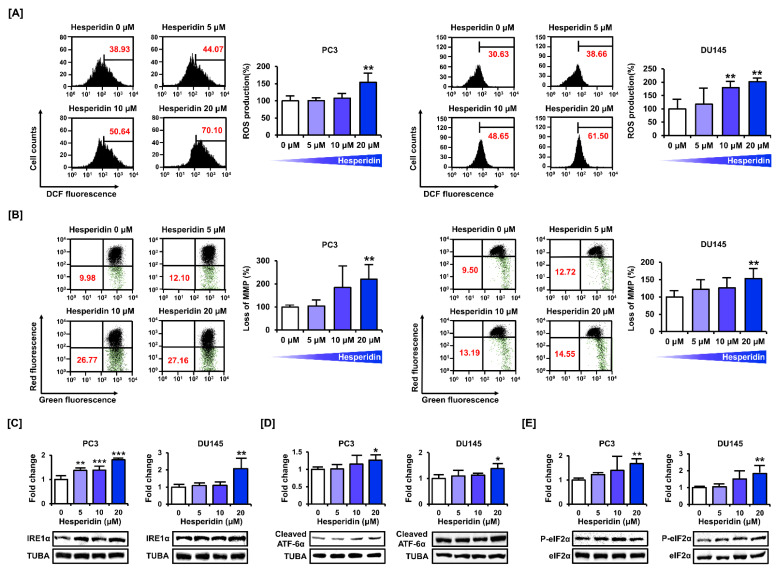
Effect of hesperidin on mitochondrial dysfunction in prostate cancer cells. (**A**) Reactive oxygen species (ROS) generation was estimated based on DCF fluorescence intensity in PC3 and DU145 cells. (**B**) Mitochondrial membrane potential (MMP) was measured in PC3 and DU145 cells after staining with JC-1 dye. (**C**–**E**) Protein expression levels of inositol-requiring enzyme 1α (IRE1α) (**C**), cleaved activating transcription factor 6α (ATF6α) (**D**), phosphorylated eukaryotic initiation factor 2α (peIF2α) (**E**) were analyzed via immunoblotting in PC3 and DU145 cells after hesperidin treatment for 24 h in a dose-dependent manner (0, 5, 10, and 20 μM). All results of the arithmetic mean (*n* = 3) denote statistical significance with asterisks (* *p* < 0.05, ** *p* < 0.01, and *** *p* < 0.001).

We measured mitochondrial homeostasis by estimating the relative levels of oxidative stress, and MMP and ER function in prostate cancer cells. Oxidative stress plays an important role in the disruption of mitochondrial function. DCF fluorescence detection indicated that hesperidin induced ROS production in PC3 and DU145 cells (Figure 3A). In addition, we assessed depolarization of the mitochondrial membrane in PC3 and DU145 cells by staining with JC-1 dye. Treatment with hesperidin increased JC-1 green monomers in PC3 and DU145 cells in a dose-dependent manner (Figure 3B). We then confirmed the expression of the proteins involved in ER stress. The expression levels of IRE1α, cleaved ATF-6α, and phosphor-eIF2α were increased after dose-dependent treatment with hesperidin in PC3 and DU145 cells (Figure 3C–E). These results indicate that hesperidin induces oxidative stress, mitochondrial homeostasis, and ER stress in prostate cancer cells.

### 3.4. Ca^2+^ homeostasis Is Important for the Effect of Hesperidin on the Proliferation of Prostate Cancer Cells

**Figure 4:**
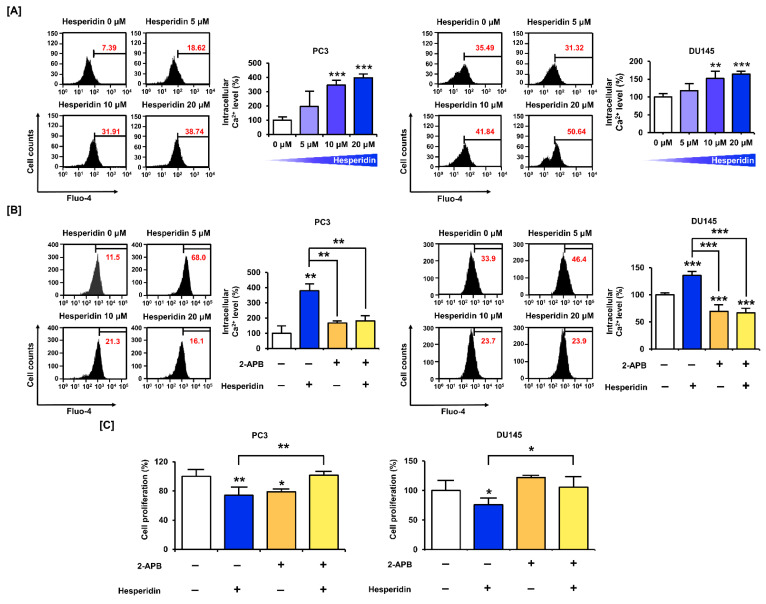
Effect of hesperidin on intracellular Ca^2+^ levels in prostate cancer cells. (**A**) Changes in intracellular Ca^2+^ concentrations after hesperidin treatment were determined via fluo-4 staining in PC3 and DU145 cells. (**B**) Changes in intracellular Ca^2+^ concentrations via co-treatment with hesperidin and 2-aminoethyl diphenyl borate (2-APB; 2 μM) were determined via fluo-4 staining in PC3 and DU145 cells. (**C**) Relative proliferation of PC3 and DU145 cells was verified after co-treatment with hesperidin and 2-APB for 48 h. All results of the arithmetic mean (*n* = 3) denote statistical significance with asterisks (* *p* < 0.05, ** *p* < 0.01, and *** *p* < 0.001).

We stained PC3 and DU145 cells with fluo-4 AM dye to measure intracellular Ca^2+^ levels following hesperidin treatment. The results showed that hesperidin increased Ca^2+^ levels in PC3 and DU145 cells in a dose-dependent manner. Compared with the control, the concentration of cytosolic Ca^2+^ increased by 3.97 folds (*p* < 0.001) in PC3 and 1.64 folds (*p* < 0.001) in DU145 cells treated with 20 µM hesperidin (Figure 4A). Additionally, the cells were co-treated with hesperidin and 2-aminoethyl diphenyl borate (2-APB), which inhibited the upregulation of Ca^2+^ in the cytoplasm. The co-treatment with 2-APB alleviated the elevation of intracellular Ca^2+^ levels induced by hesperidin treatment alone (Figure 4B). Based on these results, we investigated the changes in proliferative capacity in response to the combined treatment with hesperidin and 2-APB and hesperidin alone. Interestingly, the addition of 2-APB restored the hesperidin treatment-induced decrease in cell proliferation in PC3 and DU145 cells (Figure 4C). These results indicate that the disruption of Ca^2+^ homeostasis is an important mechanism involved in the anticancer effects of hesperidin.

### 3.5. Hesperidin Regulates Genes Related to Store Operated Ca^2+^ Entry (SOCE) in Prostate Cancer Cells

**Figure 5:**
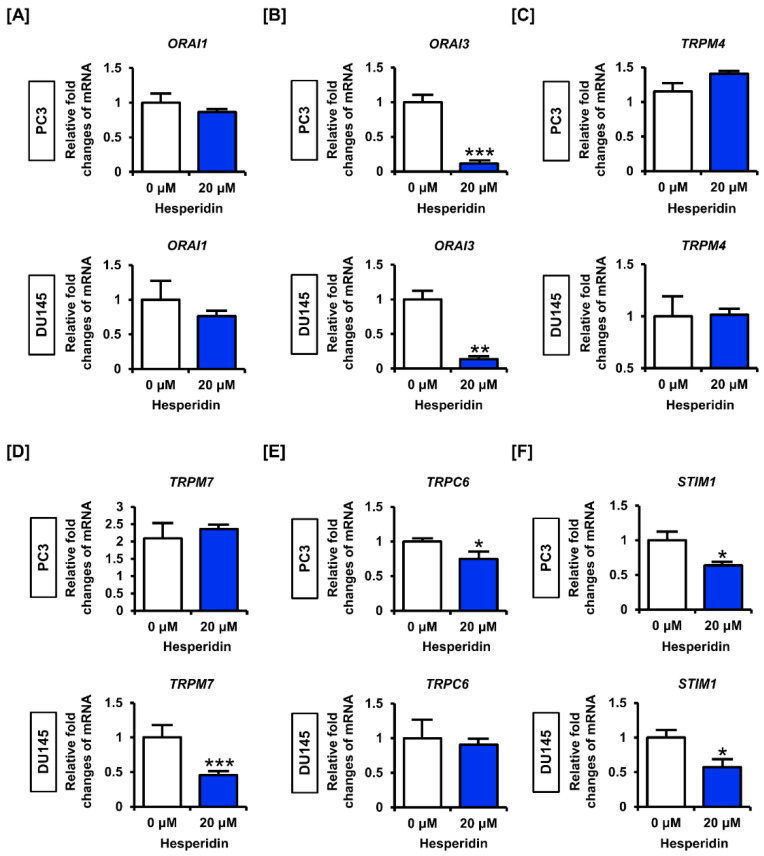
Effect of hesperidin on the expression levels of genes related to the store operated calcium entry (SOCE) in prostate cancer cells. (**A**–**F**) Expression levels of *calcium release-activated calcium modulator* (*ORAI*)-*1* (**A**), *ORAI3* (**B**), *transient receptor potential cation channel subfamily M* (*TRPM*)-*4* (**C**), *TRPM7* (**D**), *transient receptor potential cation channel subfamily C member 6* (*TRPC6*) (**E**) and *stromal interaction molecule 1* (*STIM1*) (**F**) in PC3 and DU145 cells following hesperidin treatment were determined via quantitative polymerase chain reaction (qPCR). Expression of *glyceraldehyde 3-phosphate dehydrogenase* (*GAPDH*) was used for normalization. All results of the arithmetic mean (*n* = 3) denote statistical significance with asterisks (* *p* < 0.05, ** *p* < 0.01, and *** *p* < 0.001).

Next, we analyzed the expression of genes involved in SOCE in prostate cancer cells. The results showed that hesperidin slightly decreased the expression levels of *calcium release-activated calcium modulator 1* (*ORAI1*) and significantly suppressed the expression of *ORAI3* in PC3 and DU145 cells (Figure 5A,B). However, hesperidin did not significantly affect the expression levels of *transient receptor potential cation channel subfamily M member 4* (*TRPM4*) in PC3 or DU145 cells, but suppressed the expression of *TRPM7* only in DU145 cells (Figure 5C,D). Moreover, hesperidin significantly decreased the expression levels of *transient receptor potential cation channel subfamily C member 6* (*TRPC6*) only in PC3 cells but suppressed the expression of *stromal interaction molecule 1* (*STIM1*) in both PC3 and DU145 cells (Figure 5E,F). These results suggest that hesperidin partially regulates the expression of genes related to SOCE; however, further studies are required to verify the detailed mechanisms of Ca^2+^ imbalance caused by hesperidin in prostate cancer.

### 3.6. Hesperidin Regulates the PI3K and MAPK Signaling Pathways in Prostate Cancer Cells

**Figure 6:**
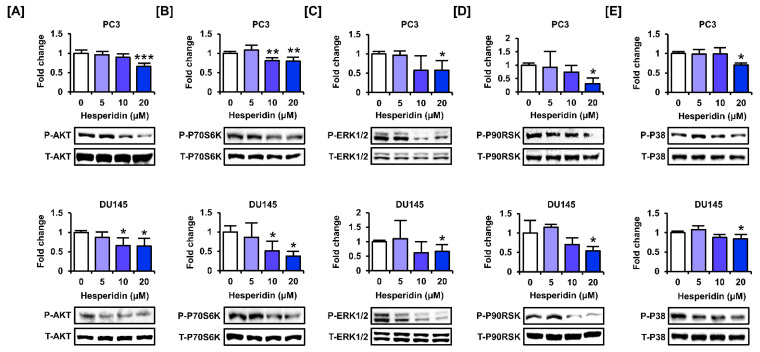
Effect of hesperidin on the expression levels of proteins involved in the phosphoinositide 3-kinase (PI3K) and mitogen-activated protein kinase (MAPK) pathways in prostate cancer cells. (**A**–**E**) Expression levels of serine-threonine kinase (AKT) (**A**), P70S6K (**B**), extracellular signal-regulated kinase (ERK)-1/2 (**C**), P90RSK (**D**), and P38 (**E**) in PC3 and DU145 cells following hesperidin treatment were determined via immunoblotting. All results of the arithmetic mean (*n* = 3) denote statistical significance with asterisks (* *p* < 0.05, ** *p* < 0.01, and *** *p* < 0.001).

We investigated whether hesperidin regulated the phosphorylation of proteins involved in the PI3K and MAPK signaling pathways, which are major signaling cascades that promote cell proliferation in cancer cells. The results showed that hesperidin inhibited the phosphorylation of AKT and P70S6K, which are involved in the PI3K pathway, in PC3 and DU145 cells (Figure 6A,B). Additionally, hesperidin suppressed the phosphorylation of ERK1/2 MAPK and its downstream protein, P90RSK, in PC3 and DU145 cells (Figure 6C,D). Moreover, hesperidin inhibited P38 MAPK at the highest concentration (20 µM) in PC3 and DU145 cells (Figure 6E). Overall, hesperidin suppressed the activation of the PI3K and MAPK signaling pathways in prostate cancer cells.

**Figure 7:**
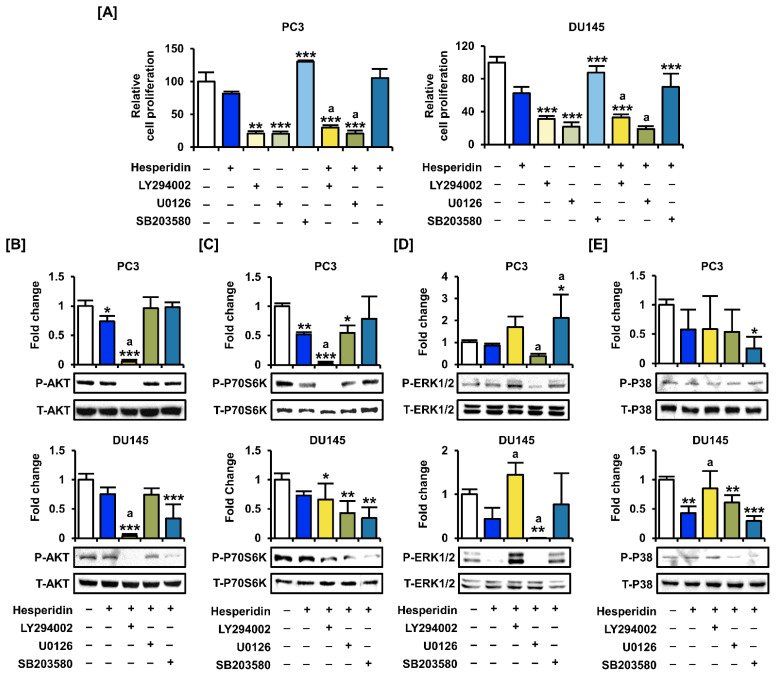
Effects of inhibitors of the PI3K and MAPK pathways on the proliferation of prostate cancer cells treated with hesperidin. (**A**) Relative proliferation of PC3 and DU145 cells was verified after cotreatment with LY294002, U0126, or SB203580 and hesperidin for 48 h. (**B**–**E**) Phosphorylation of AKT (**B**), P70S6K (**C**), ERK1/2 (**D**), and P38 (**E**) proteins in PC3 and DU145 cells following cotreatment with LY294002, U0126, or SB203580 and hesperidin was analyzed via immunoblotting. All results of the arithmetic mean (*n* = 3) denote statistical significance with asterisks (* *p* < 0.05, ** *p* < 0.01, and *** *p* < 0.001). The symbol ‘a’ indicates statistical significance (* *p* < 0.05) of the combination treatment compared to hesperidin treatment alone.

To analyze the synergistic effect of hesperidin with inhibitors of the PI3K and MAPK signaling pathways, we co-treated prostate cancer cells with LY294002 (PI3K inhibitor), U0126 (ERK1/2 inhibitor), and SB203580 (P38 inhibitor). The proliferation assay showed that co-treatment with LY294002 and U0126 significantly suppressed the proliferation of PC3 and DU145 cells compared to hesperidin treatment alone (Figure 7A). Next, we investigated changes in signaling pathways in the presence of selective inhibitors and hesperidin in prostate cancer cells. After co-treatment with selective inhibitors, the phosphorylation of AKT and P70S6K was significantly suppressed by LY294002, whereas the phosphorylation of ERK1/2 and P38 was inhibited by U0126 and SB203580 (Figure 7B–E). Moreover, the addition of LY294002 to DU145 cells contributed to increased phosphorylation of ERK1/2 and P38 proteins involved in the MAPK pathway. These results suggest that AKT may act downstream of MAPK in hesperidin-regulated signaling pathways; however, further studies are needed to elucidate the detailed mechanisms.

### 3.7. Hesperidin Induces Apoptosis with or without Cisplatin and Paclitaxel in Prostate Cancer Cells

**Figure 8:**
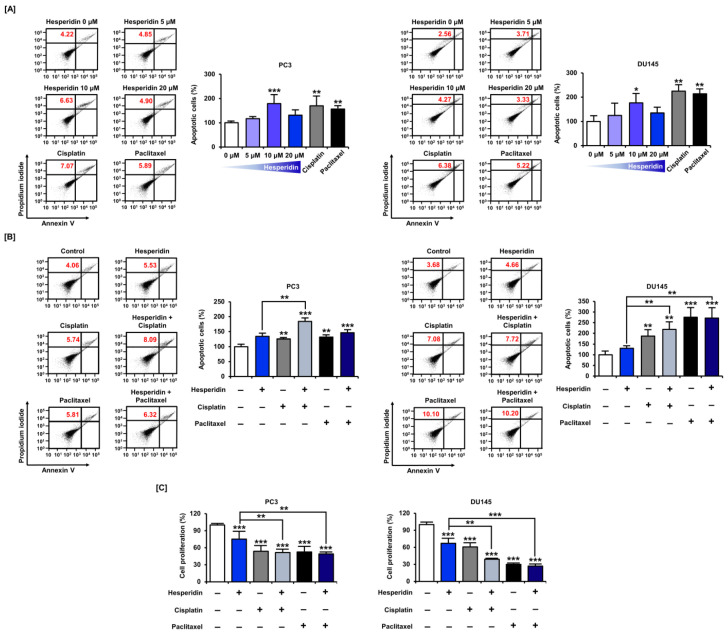
Effect of hesperidin on the apoptosis of prostate cancer cells. (**A**) Flow cytometry detection of apoptosis in PC3 and DU145 cells in response to hesperidin (0, 5, 10, and 20 μM), cisplatin (5 μM), and paclitaxel (5 μM) for 48 h. (**B**) Flow cytometry detection of apoptosis in PC3 and DU145 cells in response to combined treatment with hesperidin (20 μM) and cisplatin (5 μM) or paclitaxel (5 μM) for 48 h. The percentage of late apoptotic cells (upper right quadrant) in the treated group was compared to that in control. (**C**) Relative proliferation of PC3 and DU145 cells was verified after co-treatment with hesperidin (20 μM) and cisplatin (5 μM) or paclitaxel (5 μM) for 48 h. Statistical significance is denoted by asterisks for all results of the arithmetic mean (*n* = 3) (* *p* < 0.05, ** *p* < 0.01, and *** *p* < 0.001).

Based on the results that hesperidin increased the proportion of cells corresponding to the subG1 phase in prostate cancer cells, we analyzed whether hesperidin could induce apoptosis in these cells by staining with annexin V and PI. Similar to cisplatin and paclitaxel, 10 µM hesperidin induced a significant increase in apoptosis in both PC3 and DU145 cells (Figure 8A). Next, we investigated whether the anticancer drugs, cisplatin and paclitaxel, had any synergistic effects when co-administered with hesperidin. Apoptosis analysis revealed that the combined treatment with hesperidin and cisplatin induced a significant increase in apoptosis in PC3 cells compared to treatment with hesperidin alone (Figure 8B). Moreover, in DU145 cells, hesperidin induced a higher rate of apoptosis on co-treatment with cisplatin or paclitaxel compared to treatment with hesperidin alone. The combined anticancer effects of hesperidin and cisplatin or paclitaxel were also demonstrated in a cell proliferation assay. In PC3 and DU145 cells, co-treatment with cisplatin and paclitaxel induced a greater reduction in cell proliferation compared to treatment with hesperidin alone (Figure 8C). These results suggest that hesperidin induces apoptosis in prostate cancer cells, which is increased on co-treatment with conventional anticancer drugs.

## 4. Discussion

In this study, we investigated the physiological effects of hesperidin, a flavanone glycoside, on prostate cancer cells. Based on previous studies that showed conflicting results on the inhibition of proliferation by hesperidin in prostate cancer cells, we confirmed the antiproliferative effect of hesperidin in a dose-dependent manner [17,18]. However, hesperidin treatment did not affect the proliferation of normal prostate cells. We found that in prostate cancer cells, hesperidin induces various physiological modulations, such as ROS generation, mitochondrial disruption, regulation of ER stress-related proteins, and inactivation of signaling pathways. Among them, we focused on the contribution of hesperidin to the increase in intracellular Ca^2+^ levels, and used inhibitors of Ca^2+^ channels, suggesting that hesperidin can inhibit cell proliferation by mediating the increase in intracellular Ca^2+^ in prostate cancer. In addition, the synergistic effects of co-treatment with anticancer drugs, such as cisplatin and paclitaxel, on the induction of apoptosis and reduction in cell proliferation suggest the potential benefit of hesperidin as a therapeutic adjuvant for prostate cancer.

Prostate cancer has a high mortality rate because of acquired resistance. Despite the development of ADT, including enzalutamide and abiraterone, most patients develop castration-resistant prostate cancer [28]. Although recent genome-based studies have improved prostate cancer management owing to the advances in personalized diagnosis and treatment, castration-resistant prostate cancer still has a high mortality rate [29,30]. Nutraceutical therapy is a key approach in the development of adjuvants that can synergize with existing clinical anticancer drugs [31]. Previous studies have provided evidence that citrus extracts may contribute to the inhibition of proliferation and induction of apoptosis in cancer cells [32]. Citrus peels contain a high proportion of flavonoids and have been shown to inhibit tumorigenesis by both intraperitoneal injection and oral administration in a prostate tumor xenograft mouse model [33]. In addition, anticancer effects of various compounds extracted from citrus fruits have been reported. In hepatocellular carcinoma cells, hesperetin induces the activation of apoptosis-inducing factors by mediating the excessive production of intracellular ROS and increasing Ca^2+^ levels [34]. Nobiletin, a polymethoxyflavone present in citrus fruits, has a synergistic effect in inhibiting colony formation and inducing apoptosis of prostate cancer cells when combined with anti-androgen drugs used for prostate cancer therapy [35]. Hesperidin is also a major compound found in citrus fruits and is well known for its wide range of pharmacological effects, such as anti-inflammatory, anti-allergic, and anti-viral effects [36]. Preclinical studies have confirmed that it plays a role in preventing the progression of malignancy [37]. Hesperidin inhibits cell proliferation and induces apoptosis by modulating molecular targets and signaling pathways in various cancers. In lung cancer, hesperidin inhibits cell proliferation by targeting the miR-132/ZEB2 signaling pathway [14]. In rats, hesperidin also inhibits renal cancer progression by inhibiting inflammatory pathways and decreasing PCNA expression [38]. Moreover, numerous studies using cell and animal models have shown that hesperidin is not harmful to normal cells, tissues, and organs, including nerves, intestines, and kidneys [39,40,41]. The mechanism by which hesperidin has a specific antiproliferative effect on prostate cancer cells, without affecting the proliferation of normal cells, requires further study. We speculate that the oxidative stress-inducing effect of hesperidin may be related to its cancer-specific action. Oxidative stress, which is higher in cancer cells than in normal cells, is considered a strategy to exceed the survival threshold of cancer cells via excessive oxidative stress induction to exert anticancer effects [42].

Among the multiple functions of hesperidin in prostate cancer cells, we specifically noted the disturbance of Ca^2+^ homeostasis. Ca^2+^ regulates various cellular functions, such as fertilization, cell differentiation, proliferation, apoptosis, cell cycle, and ATP synthesis [43]. We also identified elevation of cell proliferation by 2-APB, an IP3R inhibitor, implying that an increase in intracellular Ca^2+^ concentration is important for the antiproliferative function of hesperidin in prostate cancer cells. Various Ca^2+^ channels exist in the cell membrane and are involved in the survival of prostate cancer cells. We analyzed the expression of genes related to SOCE to determine the effect of hesperidin on Ca^2+^ accumulation. ORAI is a Ca^2+^ channel subunit, and intracellular Ca^2+^ overexpression promotes ER stress-mediated apoptosis through partial reduction of the homomultimer ORAI1, IP3R, and SERCA channels [44]. Moreover, ion inflow through TRPC6 is necessary for plasma membrane localization of ORAI1. [45]. A previous study using DU145 cells reported that prostate cancer cell migration was regulated by the blockage of ORAI1 by 2-APB, enhancing the therapeutic efficacy of the Na^+^/K^+^ ATPase inhibitor [46]. TRPM4 is upregulated in various cancers, and increased intracellular Ca^2+^ activates TRPM4, leading to Na^+^ ion influx. Na^+^ ions then inhibit the ORAI1 channel through depolarization [47]. Furthermore, an increase in TRPM7 reduces the Ca^2+^/Mg^2+^ ratio, resulting in a decrease in cell proliferation [48]. In this study, hesperidin partially regulated genes involved in SOCE; therefore, further studies are required to verify the detailed mechanisms of Ca^2+^ regulation in prostate cancer.

IP3R exists in the endoplasmic reticulum and is an important receptor that affects ER stress. The ER produces lipids, synthesizes proteins, and regulates Ca^2+^ storage [49]. The ER forms the lumen, which proceeds with protein folding and maturation, and control pathways, such as UPR and autophagy, exist to maintain ER homeostasis [50,51]. We identified the increased expression of IRE1α and cleaved ATF-6α of transmembrane mediators among several transmembrane mediators present in the ER. Previous evidence suggests that tunicamycin, which induces ER stress, causes apoptosis in androgen-resistant prostate cancer cells [52]. Tunicamycin-induced apoptosis in prostate cancer cells is dependent on ROS generation and mitochondrial membrane disruption. Oxidative stress plays a key role in the initiation and progression of many types of cancers. Epidemiological, clinical, and experimental studies have suggested that oxidative cell damage may contribute to the development of prostate cancer [53]. Because of the redox imbalance caused by oxidative stress, the functions of proteins, including transcription factors sensitive to redox conditions, are regulated; thus, gene expression is also altered [54]. Changes in protein activity caused by oxidative stress generally led to the activation of proliferative signaling pathways, contributing to carcinogenesis. In particular, intracellular free radicals caused by oxidative stress promote the activation of Ca^2+^- and phosphorylation-dependent signaling pathways [55]. Paradoxically, high levels of oxidative stress, represented by an increase in ROS in cancer cells, are also targeted for therapeutic approaches such that ROS levels exceed the threshold to induce oxidative stress to an even greater extent, leading to apoptosis [7,56]. ROS are a by-product of mitochondrial respiration but also play an important role in the regulation of cell signaling pathways [57].

Activation of the PI3K and MAPK pathways is considered a key signaling pathway for cell proliferation and survival [58]. MAPK is a serine/threonine kinase family that is involved in cell proliferation. In addition, the PI3K pathway is activated by various proliferative factors, and phosphorylation of AKT is increased, leading to the stimulation of cell proliferation, survival, and differentiation to promote carcinogenesis [59,60]. AKT interacts with activated IP3R, reduces its ability to release Ca^2+^, and may reduce cellular vulnerability to apoptotic stress through pathways that include reduced Ca^2+^ mobilization between the ER and mitochondria [61]. Therefore, targeting the MAPK and PI3K pathways is regarded as the primary approach for cancer treatment. We confirmed that hesperidin inhibited the phosphorylation of proteins involved in the PI3K (AKT and P70S6K) and MAPK (ERK1/2, P90RSK, and P38) pathways in prostate cancer cells. Similar to our findings, a previous study reported that hesperidin inhibits the PI3K pathway in chemically induced liver cancer tissues in rats [62]. Hesperidin also regulates the expression of genes involved in cell cycle progression to arrest the cell cycle by inhibiting the MAPK pathway in intrahepatic cholangiocarcinoma [63]. To the best of our knowledge, this study is the first to analyze the effects of hesperidin on the MAPK and PI3K signaling pathways in prostate cancer cells.

## 5. Conclusions

**Figure 9:**
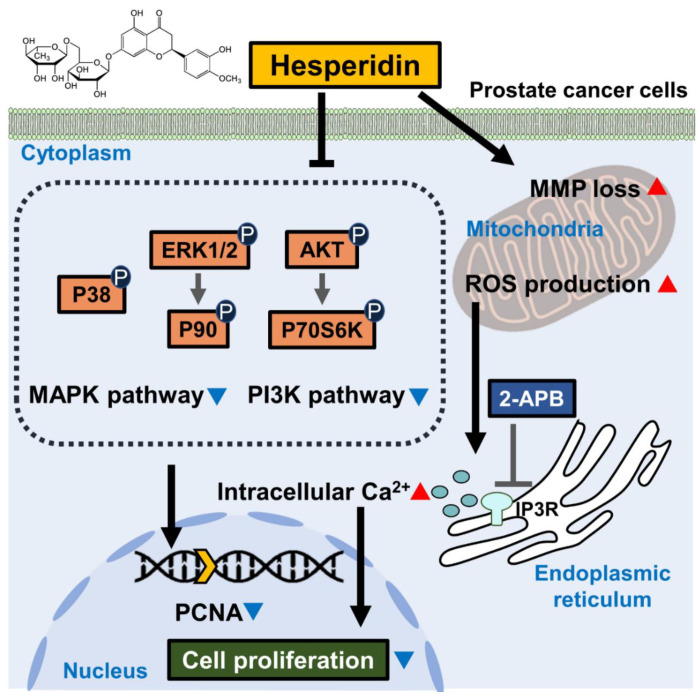
Scheme indicating the mechanism of action of hesperidin in prostate cancer cells. Hesperidin inhibits the PI3K and MAPK signaling pathways in prostate cancer cells. In addition, hesperidin induces the loss of mitochondrial membrane potential (MMP) and promotes reactive oxygen species (ROS) generation. Hesperidin also promotes the levels of Ca^2+^ released from the endoplasmic reticulum (ER), which is prevented by 2-aminoethyl diphenyl borate (2-APB). Hesperidin-induced physiological changes lead to the inhibition of cell proliferation, which is represented by a decrease in PCNA levels in the nucleus of prostate cancer cells.

Taken together, this study confirmed the inhibition of cell proliferation and colony formation by hesperidin in two castration-resistant prostate cancer cell lines (Figure 9). In addition, hesperidin induced oxidative stress and mitochondrial membrane disruption in prostate cancer cells. It is speculated that hesperidin-induced increase in intracellular Ca^2+^ levels in prostate cancer contributes to its antiproliferative effect. This study also verified that PI3K and MAPK pathway activation was inhibited by hesperidin treatment in prostate cancer cells, suggesting that hesperidin can potentially be used as a therapeutic adjuvant for prostate cancer. Hence, further studies are needed to verify the therapeutic value of hesperidin.

## Figures and Tables

**Table 1:** Primer sets used in qPCR.

Gene Symbol	Sense Primer (5′→3′)	Antisense Primer (5′→3′)
*ORAI1*	ACGTGCACAATCTCAACTCG	AGAACTTGACCCAGCAGAGC
*ORAI2*	GAGCAACATCCACAACCTGA	GCTGCTCTGCTGGATCAAGT
*TRPM4*	CTGCATCGACTTCATGGTTT	CGTGAGCAAGATGATGAAGG
*TRPM7*	AAGATCTTTCAGCCCTGACG	GCATTTCCAAACACTTGGCT
*TRPC8*	GAACTTCCGAAGAGGCTTCC	GCAAGCTCTCTTCATCTGGG
*SIM1*	GACCCAGACACACCATCTCC	GCTGTGGCTGAGGAGGATAA
*GAPDH*	GGCTCTCCAGAACATCATCC	TTTCTAGACGGCAGGTCAGG

## Data Availability

Data are contained within this article.
